# Mutation of *Isocitrate Dehydrogenase 1* in Cholangiocarcinoma Impairs Tumor Progression by Inhibiting Isocitrate Metabolism

**DOI:** 10.3389/fendo.2020.00189

**Published:** 2020-04-21

**Authors:** Li Su, Xinglong Zhang, Lei Zheng, Miaomiao Wang, Zhifa Zhu, Ping Li

**Affiliations:** ^1^Department of Integrated Traditional and Western Medicine in Oncology, First Affiliated Hospital of Anhui Medical University, Hefei, China; ^2^Anhui University of Traditional Chinese Medicine, Hefei, China

**Keywords:** cholangiocarcinoma, isocitrate dehydrogenase 1, aldehyde dehydrogenase 1, α-ketoglutarate, NADPH

## Abstract

**Aim:** Isocitrate dehydrogenase 1 (IDH1) is key enzyme involved in cellular metabolism and DNA repair. Mutations in IDH1 occur in up to 25% of cholangiocarcinomas. The present study aimed to explore the features of cellosaurus REB cells with mutant and wide-type *IDH1*.

**Methods:** To compare the features of *IDH1* knockout and mutation in cholangiocarcinoma, we firstly constructed the *IDH1* knockout and *IDH1* mutation cell lines. We then evaluated the viability of these cell lines using the cell count assay and MTT assay. Next, we determined cell migration and invasion using the Transwell assay. Additionally, to evaluate the effects of IDH1 on cellular metabolism, the levels of α-ketoglutarate (α-KG) and nicotinamide adenine dinucleotide phosphate (NADPH) were determined using enzyme-linked immunosorbent assay. We then applied ChIPbase dataset to explore the genes that were regulated by *IDH1*.

**Results:** High frequency of mutated *IDH1* was observed in the cholangiocarcinoma and *IDH1* R132C was presented in more than 80% of mutations. The results showed that *IDH1* knockout decreased cell proliferation, migration and invasion, whereas the overexpression of *IDH1* in *IDH1* knockout cell line recovered its proliferation, migration and invasion capacities. Additionally, *IDH1* mutation reduced the levels of NADPH and α-KG. Furthermore, investigation into the underlying mechanisms revealed that *IDH1* overexpression induced the expression of aldehyde dehydrogenase 1 thereby promoting cell proliferation, migration and invasion.

**Conclusion:** *IDH1* plays an important role in cholangiocarcinoma and its mutation impairs tumor progression in part by inhibition of isocitrate metabolism.

## Introduction

Cholangiocarcinoma is an adenocarcinoma of epithelial cells that occurs in any areas of bile duct (1). Although it is a rare type of cancer, its occurrence has increased in the past three decades (2). Cholangiocarcinoma can be classified into intrahepatic, perihilar and distal types based on their clinical features. However, surgery and liver transplantation are the recommended therapeutic options for all types of cholangiocarcinoma in a minority of patients (1, 3). The 5-years survival rate is 15% for patients with early-stage intrahepatic cholangiocarcinoma. However, if the intrahepatic cholangiocarcinoma has spread to a distant part of the body, the 5-years survival rate is reduced to only 2% (4). Many patients are diagnosed with cholangiocarcinoma in an advanced stage. Due to the fact that surgery cannot completely remove metastatic tumors, additional treatment is recommended (2, 5).

Genetic alternations are thought to play a critical role in the occurrence and development of cholangiocarcinoma (2, 6, 7). Mutations in several oncogenes and tumor suppressor genes have been reported to be involved in the carcinogenesis of cholangiocarcinoma (6). For instance, activated *KRAS* has been reported to be associated with the development of cholangiocarcinoma (8). Inactivated p53, a tumor suppressor gene, is observed in the tumor tissue of cholangiocarcinoma (8, 9). In addition to those genes, other genes including *SMAD4, PTEN, EGFR*, and *PDGFB* have also been described to be associated with the occurrence and development of cholangiocarcinoma (10).

Isocitrate dehydrogenase 1 (IDH1) is an enzyme encoded by *IDH*1, which is responsible for catalyzing isocitrate to produce α-ketoglutarate (α-KG) and generating nicotinamide adenine dinucleotide phosphate (NADPH) (11, 12). α-KG and NADPH are components of the tricarboxylic acid (TCA) cycle, which are thought to detoxify against oxidative stress (12, 13). Therefore, IDH1 indirectly regulates oxidative damage. Additionally, IDH1 has also been reported to play an important role in the process of glucose and lipid metabolism (14). Recently, alternations of *IDH1* have been implicated in many types of cancer (11). In 2008, for the first time, Parsons and colleagues have demonstrated *IDH1* mutations in the human genome related to the glioblastoma multiforme (15). The following studies performed by other groups have further revealed that mutations in *IDH1* are associated with leukemia, colon cancer and prostate cancer (11, 12, 14). In 2012, Borger and colleagues have revealed *IDH1* mutations in cholangiocarcinoma (16). Interestingly, mutations in *IDH1* have been frequently observed in poorly differentiated tumors (16). These results support that *IDH1* might be used as a potential biomarker for the detection of cholangiocarcinoma. In 2018, Khurshed et al. have reported that mutations in *IDH1* are associated with improved response to irradiation and chemotherapy in colon carcinoma and glioblastoma cells (17). More recently, they found that *IDH* mutation in gliomas depended on lactate and the neurotransmitter glutamate as metabolic substrates to rescue cells from the metabolic stress (18). These results suggested that *IDH* mutation might affect tumor progression by regulating metabolic stress. In the present study, we aimed to explore the effects of *IDH1* mutation on cholangiocarcinoma. Furthermore, we revealed the mechanisms of *IDH1* mutation underlying the tumor progression of cholangiocarcinoma.

## Materials and Methods

### Cell Line and Cell Viabilities

Cholangiocarcinoma RBE cell line was purchased from the First Affiliated Hospital of Anhui Medical University and cultured in complete Dulbecco modified eagle medium (DMEM) containing 10% fetal bovine serum (FBS) and 1% antibiotics under 37°C in the presence of 5% CO_2_ at constant humidity.

Cell viability of RBE cell line and RBE IDH1 knockout or mutation cells was determined using the MTT assay and cell count assay. For MTT assay, an MTT solution (Sigma, St. Louis, MO, USA) was added into each well and the plate was incubated at 37°C. After 4 h, DMSO solution was added and the optical density was read at 570 nm using a microplate reader (Molecular Devices, Sunnyvale, CA, United States). For cell count assay, trypan blue staining solution was added to the cells and then the cell viabilities were calculated by counting live and dead cells.

### Construction of IDH1 Knockout and IDH1 Mutation Cell Line

The IDH1 knockout (IDH1 KO) cell line was constructed using CRISPR-Cas9 (Shanghai Liangtai Biotech Company, Shanghai, China). In brief, when the IDH1 cells reached 70% confluency, the cells were transfected with CRISPR-Cas9 knockout plasmids containing guide RNA sequence of IDH1 and sequence of Cas9 protein.

The IDH1 R132C mutation cell line was constructed by transfecting the IDH1 KO cell line with IDH1 R132C mutation plasmids.

### Cell Invasion and Migration Assays

Cell invasion and migration assays were performed according to previously reported methods (19, 20). Transwell chamber consisted of a membrane filter coated with Matrigel was used in this study. In brief, the cells were detached using trypsin and then resuspended in serum-free DMEM medium. The cells were then seeded into the upper chamber and the complete DMEM medium was added into the lower chamber. The invaded cells which located in lower chamber were counted after incubation for 12 h. Crystal violet solution was used to stain the cells and then a microscope was used to count the cells.

### Detection of α-KG and NADPH

The levels of α-KG and NADPH were determined using relevant qualification kits, according to the documents of the manufacturer (BioVision, Milpitas, CA, United States).

### Xenograft Mouse Model

Male BALB/c nude mice (6 weeks old) were purchased from Guangzhou Laboratory Animal Center (Guangzhou, China). The mice were housed in a room on a 12-h light-dark cycle and fed under experimental conditions with a temperature of 22–24°C and humidity of 50 ± 5%. Animal procedures used in this study were approved by the First Affiliated Hospital of Anhui Medical University' Animal Care and Use Committee.

After 1 week acclimatization, the mice were subcutaneously injected with 2 × 10^6^ REB IDH1 KO cells, or REB IDH1 KO cells transfected with wild-type IDH1 or IDH1 R132C mutation plasmids. After 1 week, the tumor volume was measured every 3 days, according to the formula: length × width^2^ × 0.52.

### Immunohistochemistry

After the mice were sacrificed, the tumor tissue was removed and washed with cold PBS, then fixed in 4% paraformaldehyde. The tissues were embedded into paraffin and the sections were then cut at 4-μm thickness. Ki67 (KeyGEN BioTECH, Nanjing, China) staining was performed according to a previously reported method (21) and the sections were then observed under microscopy.

### Quantitative Polymerase Chain Reaction (qPCR)

Trizol reagent was used to extract total RNA from the cells, according to the instructions of the manufacturer (TaKaRa, Dalian, China). Isolation of total RNA was performed under sterile condition. To remove DNA contamination, RNase-free DNase I was used. Primers for CDH1, TJP1, VIM, FN1, Snail, Twist, ZEB1, and β-actin were used for amplification of these genes. To analyze the accuracy of the PCR reaction, the melt curves were used. To evaluate the expressions of genes, 2^−ΔΔCt^ values were calculated. The mRNA expression values of CDH1, TJP1, VIM, FN1, Snail, Twist, and ZEB1 were normalized to that of β-actin.

### Western Blotting

Extraction and qualification of proteins were performed according to a previously reported method (22). Radioimmunoprecipitation buffer was used to lysis the cells. Next, the lysate was centrifuged at 13,000 *g* to remove insoluble materials. The concentrations of protein were qualified using the BCA protein assay kit (Beyotime, Jiangsu, China).

Protein samples were loaded at equal amounts for each sample and 10% sodium dodecyl sulfate (SDS) gel was used to separate the samples. Next, the SDS gel was then transferred to a polyvinylidene fluoride membrane and 5% non-fat milk was used to block the membrane. The membrane was probed with primary antibody followed by an appropriated secondary antibody. An imaging system (Bio-Rad, CA, USA) was used to examine chemiluminescence. Expression values of protein were normalized to that of β-actin.

### Statistical Analysis

The TCGA cholangiocarcinoma dataset (MSK, Clin Cancer Res 2018) was downloaded and analyzed in cBioportal (www.cbioportal.org). SPSS 13.0 (SPSS, Chicago, IL, USA) was used to statistical analysis. All Data were expressed as mean ± standard deviation (S.D.). To evaluate the significance, one-way analysis of variance with multiple comparisons and Student-Newman-Keuls (SNK) test were performed. A *P* < 0.05 was considered as a statistical significance.

## Results

### IDH1 Mutation and Frequency in Cholangiocarcinoma

**Figure 1:**
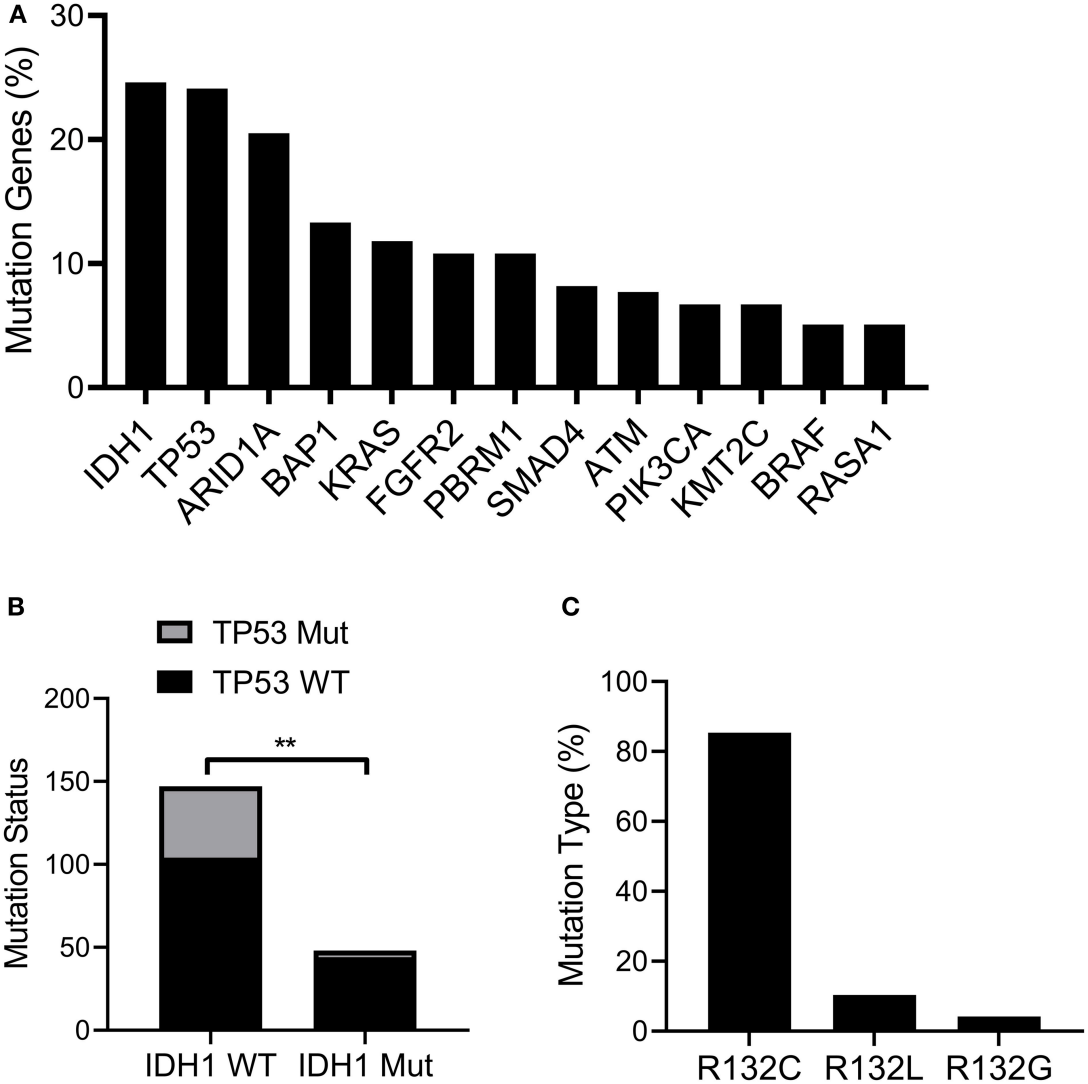
IDH1 was mutated in cholangiocarcinoma. The TCGA cholangiocarcinoma dataset (MSK, Clin Cancer Res 2018) was downloaded and analyzed in cBioportal (www.cbioportal.org). **(A)** The frequency of mutated genes in Cholangiocarcinoma. **(B)** The proportion of TP53 mutated patients in IDH1 wildtype (WT) or mutated group. **(C)** The proportion of IDH1 with different mutation types. The data were represented as mean ± S.D. **P < 0.01.

First, we explored the frequency of the mutated genes in cholangiocarcinoma. The results indicated that *IDH1* was mutated at the highest frequency among all mutated genes including TP53, ARID1A, BAP1, and KRAS (Figure 1A). We also noticed that mutated TP53 also appeared at a high frequency. It is known that TP53 mutations are frequently observed in the occurrence and development of many types of cancer and are implicated to be an important risk factor for tumorigenesis (23). Therefore, we further analyzed the frequency of mutated TP53 in patients with wild-type (WT) or mutated *IDH*. Interestingly, we found mutated TP53 with higher frequency in patients with WT *IDH* than patients with mutated *IDH* (Figure 1B). Additionally, we also detected the proportions of different mutation types of *IDH1*. The results showed *IDH1* R132C was presented in more than 80% of mutation types (Figure 1C).

### IDH1 Promoted Cell Proliferation of Cholangiocarcinoma

**Figure 2:**
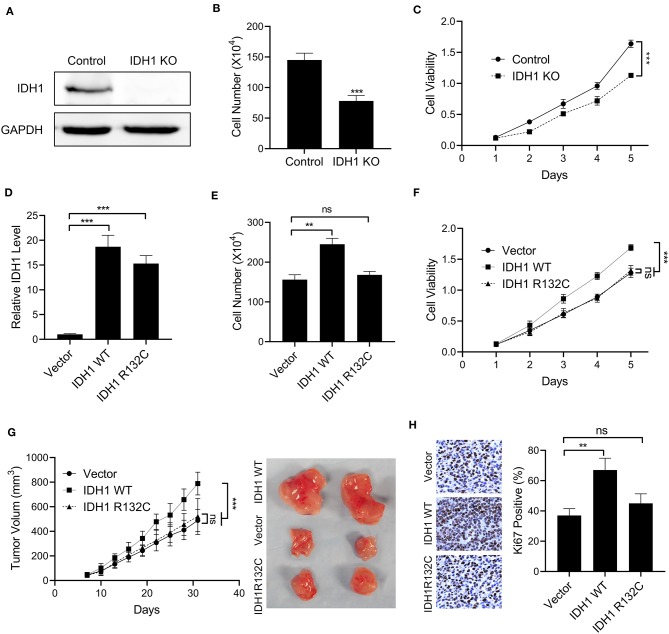
The overexpression of IDH1 promoted cell proliferation of cholangiocarcinoma. **(A)** Western blot was used to determine the expressions of IDH1 in the IDH knockout (KO) REB cells. Cell count assay **(B)** and MTT assay **(C)** were used to determine the cell viabilities of REB and IDH1 KO REB cells. **(D)** qPCR was used to determine the relative expressions of IDH1 in IDH1 KO REB cells transfected with vector, plasmids containing wildtype IDH1 or plasmids containing IDH1 R132C mutation sequence. Cell count assay **(E)** and MTT assay **(F)** were used to determine cell viabilities of IDH1 KO cells transfected with vector, plasmids containing wildtype IDH1 or plasmids containing IDH1 R132C mutation type. **(G)** The tumor volume of xenograft mouse transplanted with IDH1 KO REB cells transfected with vector, wildtype IDH1 or IDH1 R132C mutation plasmid. **(H)** Immunohistochemistry demonstrated percentage of Ki67 positive in tumor tissue from each group. Data are shown as mean ± S.D. **P < 0.01, ***P < 0.001; ns indicates no significance.

To explore the effects of *IDH1* on cell proliferation, the *IDH1* KO and WT cell lines were constructed. The IDH1 WT cell line was created in the *IDH1* KO cells transfected with WT *IDH1* expressing plasmid. As shown in Figure 2A, we did not find expression of *IDH1* in the *IDH1* KO cells, indicating *IDH1* was successfully knocked out. Cell count assay and MTT assay demonstrated that cell number and viability were significantly decreased after *IDH1* was knocked out (Figures 2B,C). Next, the IDH1R132C cell line was created in the IDH1 KO cells with IDH1 R132C expressing plasmid. As shown in Figure 2D, the *IDH1* R132C mutation and the *IDH* WT cells were successfully constructed. The results showed that cell number and viability were significantly increased in the *IDH* WT cells when compared with those in cells transfected with vector control (Figures 2E,F). However, we did not find a significant difference in cell number and viability between *IDH* R132C mutation cells and cells transfected with vector control. Furthermore, a xenograft animal model was constructed. As shown in Figure 2G, tumor volume in animals transplanted with the *IDH* WT cells were significantly increased when compared with that in animals transplanted with the IDH R132C mutation cells or cells transfected with vector control. Additionally, we found more Ki67 positive cells in tumor tissues from animals transplanted with the *IDH* WT cells (Figure 2H).

### IDH1 Promoted Cell Migration and Invasion of Cholangiocarcinoma

**Figure 3:**
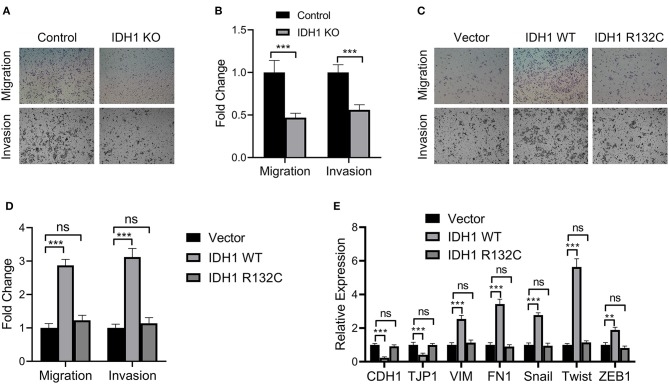
IDH1 promoted cell migration and invasion and IDH1 knockout decreased cell migration and proliferation. **(A)** Cell migration and invasion of the REB and IDH1 KO REB cells were determined using Transwell and **(B)** the values in each group were shown as mean ± S.D. **(C)** Cell migration and invasion of the IDH1 KO REB cells transfected with vector, wildtype IDH1, or IDH1 R132C mutation plasmid and **(D)** the values in each group were shown as mean ± S.D. **(E)** qPCR was used to analyze the relative expressions of a panel of EMT relevant markers in IDH1 KO REB cells transfected with vector, wildtype IDH1 or IDH1 R132C mutation plasmid. Data were shown as mean ± S.D. **P < 0.01, ***P < 0.001; ns indicates no significance.

We then investigated the effects of *IDH1* on cell migration and invasion. In the *IDH1* KO cells, we found significant reductions in cell migration and invasion (Figures 3A,B). However, in the cells transfected with plasmids containing WT *IDH1*, we observed increased cell migration and invasion when compared with those in the cells transfected with vector control (Figures 3C,D). Additionally, no significant difference for cell migration and invasion between cells transfected with plasmids containing *IDH1* R132C and cells transfected with vector control (Figures 3C,D). Moreover, the results showed the relative expression of a panel of epithelial-mesenchymal transition (EMT) markers including CDH1, TJP1, VIM, FN1, Snail, Twist, and ZEB1 were significantly changed after the cells were transfected with plasmids containing wild-type *IDH1*. We did not find cells transfected with plasmids containing *IDH1* R132C with those changes when compared with cells transfected with vector control (Figure 3E).

### IDH1 Mutation Reduced Levels of α-KG and NADPH

**Figure 4:**
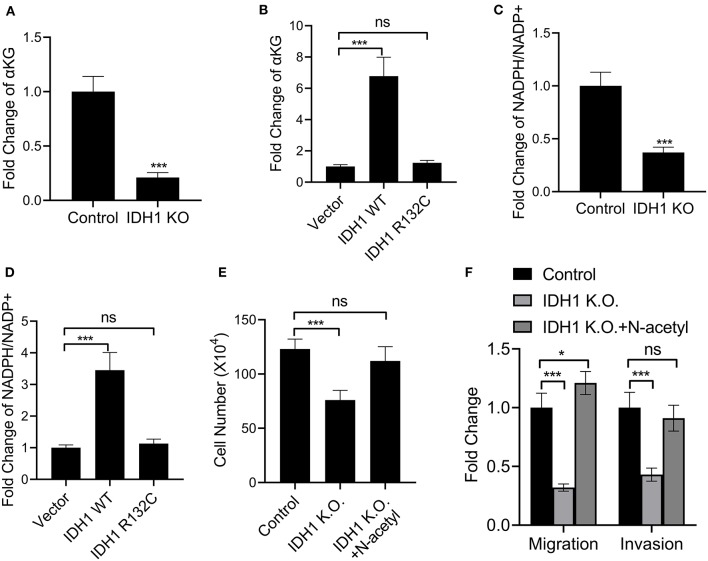
IDH1 mutation reduced NADPH and α-KG levels. **(A,B)** ELISA was used to determine the levels of α-KG in REB cells, IDH1 KO REB cells, IDH1 KO REB cells transfected with vector, wildtype IDH1 or IDH1 R132C mutation plasmid. **(C,D)** ELISA was used to determine the levels of NADPH in REB cells, IDH1 KO REB cells, IDH1 KO REB cells transfected with vector, wildtype IDH1 or IDH1 R132C mutation plasmid. **(E)** Cell count assay were used to determine the cell viabilities of REB cells or REB IDH1 K.O. cells treated with or without N-acetyl cysteine. **(F)** Cell migration and invasion assay of the REB cells or REB IDH1 K.O. cells treated with or without N-acetyl cysteine. Data were shown as mean ± S.D. *P < 0.05, ***P < 0.001; ns indicates no significance.

We next explored the mutation effects of *IDH1* on α-KG and NADPH. First, we determined the levels of α-KG and NADPH in RBE cells and *IDH* KO RBE cells. The results demonstrated that the levels of α-KG and NADPH were significantly decreased in the *IDH* KO RBE cells when compared with those in the RBE cells (Figures 4A,C). However, in the *IDH* KO cells transfected with plasmids containing WT *IDH1*, the levels of α-KG and NADPH were increased, indicating *IDH* affected the levels of α-KG and NADPH (Figures 4B,D). Interestingly, when we transfected the IDH KO cells with plasmids containing *IDH1* R132C, reductions in α-KG and NADPH were observed (Figures 4B,D). Moreover, N-acetyl cysteine treatment could significantly rescue the cell loss and impairments in migration and invasion in the IDH1 KO cells (Figures 4E,F). These results supported that *IDH1* mutation reduced levels of α-KG and NADPH.

### IDH1 Regulated the Expressions of ALDH1 in Cholangiocarcinoma

**Figure 5:**
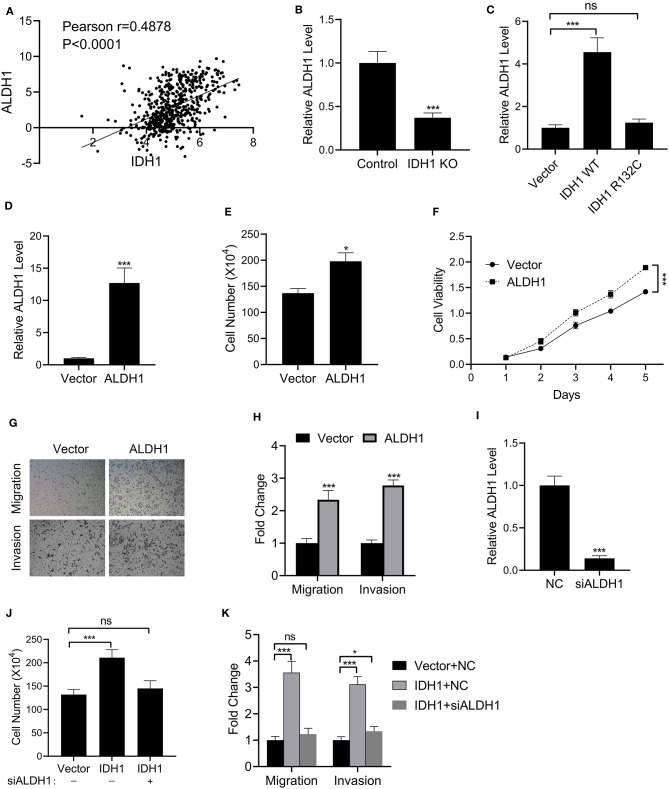
IDH1 regulated the expressions of ALDH1. **(A)** The correlation of IDH1 and ALDH1 expression in the patients with cholangiocarcinoma was analyzed using ChIPbase dataset. **(B,C)** qPCR was used to determine the levels of ALDH1 in REB cells, IDH1 KO REB cells, and IDH1 KO REB cells transfected with vector, wildtype IDH1 or IDH1 R132C mutation plasmid. **(D)** qPCR was used to determine the relative expressions of ALDH1 in REB cells transfected with ALDH1 expression plasmid. **(E,F)** Cell count assay and MTT assay were used to determine the cell viabilities of REB cells transfected with vector or ALDH1 expression plasmid. **(G,H)** Cell migration and invasion assay of REB cells transfected with vector or ALDH1 expression plasmid were determined using Transwell and the values in each group were shown as mean ± S.D. **(I)** qPCR was used to determine the relative expressions of ALDH1 in REB cells transfected with ALDH1 expression plasmid. **(J)** Cell count assay were used to determine the cell viabilities of REB cells transfected with vector or IDH1 expression plasmid in the presence or absence of ALDH1 siRNA. **(K)** Cell migration and invasion assay of the REB cells transfected with vector or IDH1 expression plasmid in the presence or absence of ALDH1 siRNA. *P < 0.05, ***P < 0.001; ns indicates not significance.

We further investigated the effects of *IDH1* on ALDH1. First, we found that IDH was correlated to the expression of ALDH1 in patients with cholangiocarcinoma (Figure 5A). We then found that The levels of ALDH1 were significantly decreased in IDH KO RBE cells (Figure 5B). Additionally, the levels of ALDH1 were significantly increased in *IDH* KO cells transfected with plasmids containing WT *IDH1*, whereas a reduction in ALDH1 was observed in the *IDH* KO cells transfected with plasmids containing *IDH1* R132C (Figure 5C). Next, we successfully transfected the *IDH* KO cells with plasmids containing ALDH1 (Figure 5D). Interestingly, the cell number and viability were increased in the *IDH* KO cells transfected with plasmids containing ALDH1 (Figures 5E,F). We also observed increased cell migration and invasion in the *IDH* KO cells with plasmids containing ALDH1 (Figures 5G,H). To further confirm if IDH1 regulated ALDH1 in such events, ALDH1 siRNA was employed to knock down the level of ALDH1 in REB cells (Figure 5I). It was found that ALDH1 siRNA could obviously reduce the viability of REB cells transfected with vector control or IDH1 expression plasmid (Figure 5J), as well as significantly decrease cell migration and invasion (Figure 5K).

## Discussion

In the present study, for the first time, *IDH1* was identified as a high frequency mutated gene in patients with cholangiocarcinoma. In our *in vitro* study, IDH promoted cell proliferation, invasion and migration, whereas those cellular events were inhibited in the IDH R132C mutation cells. In the *in vivo* study, tumor volume in mice transplanted with the *IDH* WT cells was significantly increased when compared with that in mice transplanted with the *IDH* R132C mutation cells. And more Ki67 positive cells were observed in tumor tissue from animals transplanted with the *IDH* WT cells. We further revealed that *IDH1* mutation reduced the levels of α-KG and NADPH, and *IDH1* regulated the expressions of ALDH1 in cholangiocarcinoma. These results supported that *IDH1* promoted the development of cholangiocarcinoma in part by inhibiting isocitrate metabolism.

*IDH1* mutations have been identified in many types of cancers including glioma, hepatoma, leukemia, colon cancer and prostate cancer (15, 16, 24). As a key metabolic enzyme, IDH1 is responsible for catalyzing isocitrate to produceα-KG and generating NADPH (11, 24). In the present study, we identified a high frequency of mutated IDH1 in cholangiocarcinoma.

Besides, we also found a high frequency of TP53 mutation in patients with *IDH* WT, whereas low frequency of TP53 mutation in patients was accompanied with mutated *IDH*. Adam and colleagues have demonstrated that *IDH* mutation occurs earlier in the development of glioma and its mutation precedes *TP53* mutation (25). Therefore, we speculate that *IDH* mutation and *TP53* mutation occur at different stages in cholangiocarcinoma. By analyzing the proportions of different mutation types of *IDH1*, we revealed that *IDH1* R132C was presented in more than 80% of mutation types. These results are consistent with a previous finding, in which *IDH1* mutation was found in 16.88% of patients with cholangiocarcinoma and *IDH1* R132C was presented in 12.55% of patients (26).

In the present study, we further verified the effects of *IDH1* on cholangiocarcinoma. *IDH1* promoted cell proliferation, migration and invasion in REB cells, whereas those cellular events were not significantly changed in the *IDH1* R132C REB cells. Furthermore, we found that IDH1 regulated the expression of EMT markers including CDH1, TJP1, VIM, FN1, Snail, Twist, and ZEB1. These results suggested that *IDH1* promoted the development of cholangiocarcinoma and its mutation impaired the progression of cholangiocarcinoma. Many previous studies have demonstrated that *IDH1* plays an important role in the cellular redox state in part by regulating cellular α-KG and NADPH generation within the TCA cycle (27). It is well-known that TCA cycle is essential for cell growth, because many intermediates are required for production of lipids, proteins and nucleic acids (28). Additionally, NADPH production in cancer cell mitochondria is critical to maintain glutathione and other scavenging molecules in a reduced state, thereby preventing oxidative damage to mitochondrial structures (29). However, the accumulation of α-KG and NADPH may activate cancer-related proteins including mammalian target of rapamycin 1, thereby promoting tumorigenesis (30). Regulation of α-KG levels is also essential in modulating the epigenetic landscape of cancer cells (13). In our study, the levels of α-KG and NADPH were increased in the *IDH* KO cells transfected with plasmids containing WT *IDH1*. However, when we transfected the IDH KO cells with plasmids containing *IDH1* R132C, reduced α-KG and NADPH were observed. Therefore, we verified that *IDH1* was associated with the levels of α-KG and NADPH in cholangiocarcinoma. The mutations in IDH1 affected the expression of α-KG and NADPH, thereby impacting mitochondrial functions in cholangiocarcinoma.

ALDH1 is a group of detoxifying enzymes capable of catalyzing aldehydes into carboxylic acids (31, 32). It is also a marker of stem cells and is widely expressed in many types of cancers including gastric cancer, ovarian cancer and lung cancer (33, 34). Additionally, ALDH1 is involved in cell differentiation and detoxification [32; Our results demonstrated that IDH1 regulated the levels of α-KG and NADPH, which are thought to detoxify against oxidative stress. Interestingly, we also revealed that IDH was correlated with the expression of ALDH1 in patients with cholangiocarcinoma by analyzing the ChIPbase dataset. In our *in vitro* study, the levels of ALDH1 were significantly increased in *IDH* KO cells transfected with plasmids containing WT *IDH1*, whereas a reduction in ALDH1 was observed in the *IDH* KO cells transfected with plasmids containing IDH1 R132C. We further evaluated the cellular functions including cell proliferation, migration and invasion. Interestingly, in the cells transfected with ALDH1 expression plasmids, cellular functions including cell proliferation, migration and invasion were recovered. These results supported that the effects of *IDH* on cholangiocarcinoma was mediated in part by regulating ALDH1.

## Conclusion

The present study has identified *IDH1* as a high frequency mutated gene in patients with cholangiocarcinoma. In our *in vitro* study, *IDH* promotes cell proliferation, invasion and migration. In the *in vivo* study, *IDH* increases the tumor volume in mice transplanted with the *IDH* WT cells. Further study reveals that *IDH1* regulates the levels of α-KG and NADPH and the expression of ALDH1. These results support that *IDH1* promotes the development of cholangiocarcinoma in part by inhibiting the isocitrate metabolism.

## Data Availability Statement

The raw data supporting the conclusions of this article will be made available by the authors, without undue reservation, to any qualified researcher.

## Ethics Statement

Animal procedures used in this study were approved by the First Affiliated Hospital of Anhui Medical University' Animal Care and Use Committee.

## Author Contributions

PL conceived and designed research and wrote the manuscript. LS, XZ, LZ, MW, and ZZ conducted experiments and analyzed data. All authors read and approved the manuscript.

## Conflict of Interest

The authors declare that the research was conducted in the absence of any commercial or financial relationships that could be construed as a potential conflict of interest.
